# The extended functional practical examination (EFPE): an innovative model for advancing assessment for learning in postgraduate medical education

**DOI:** 10.1186/s12909-026-10345-0

**Published:** 2026-10-02

**Authors:** Wagdy Talaat, Nourhan F. Wasfy, Enjy Abouzeid

**Affiliations:** 1 Medical Education Department, Faculty of Medicine, Suez Canal University, 41522 Round Road, Ismailia, Egypt; 2 The Egyptian Society for Medical Education (ESME), Ismailia, Egypt; 3 Faculty of Medicine, Ulster University, Londonderry, UK

**Keywords:** Extended functional practical exam (EFPE), Postgraduate assessment, Practical exam, Longitudinal assessment, Learning by doing

## Abstract

**Background:**

The Extended Functional Practical Exam (EFPE) is an innovative postgraduate assessment model designed to overcome the limitations of traditional single-point practical examinations by adopting a longitudinal, experiential, and institution-based approach. This study aimed to critically explore the implementation of EFPE across different medical schools and examine its perceived educational value, impact on learners and institutions, and areas for improvement.

**Methodology:**

A reflective qualitative study involving different medical schools was conducted. Three samples were included in the study: a convenience sample of the postgraduate students and a purposive, convenience sample of faculty members and medical school stakeholders. Insights from the three samples were collected through semi-structured interviews. All the interviews were audio recorded and transcribed verbatim and thematically analyzed.

**Results:**

The thematic analysis of data from the three samples (S1, S2, and S3) under the classified themes revealed several themes and subthemes. The main themes included the format and differences from traditional exams, the exam’s impact, and challenges and areas for improvement. Participants appreciate the alignment of EFPE tasks with institutional needs and the positive role of feedback and mentorship. However, challenges such as stress, workload, resource demands, and issues with topic selection and clarity were noted.

**Conclusion:**

EFPE is highly valued for its practical, structured, and process-focused approach, which leads to significant skill development and confidence building among postgraduate students. Suggestions from participants include extending the duration of the EFPE, involving students in topic selection, maintaining continuous feedback and mentorship, and ensuring tasks are relevant and sustainable.

**Supplementary Information:**

The online version contains supplementary material available at https://doi.org/10.1186/s12909-026-10345-0.

## Introduction

As assessment drives learning, developing better assessment tools ultimately improves learning and helps achieve the objectives of medical education. The Extended Functional Practical Exam (EFPE) emerges as a novel postgraduate assessment method specifically designed to address the limitations of traditional postgraduate practical examinations, particularly their focus on single-point evaluation and limited opportunities for feedback and authentic performance assessment [1]. The EFPE operationalizes several key assessment principles by integrating programmatic assessment, longitudinal assessment (LA), and the recommendations of the Final Ottawa 2020 Consensus Statement within a single educational model [2].

Rather than relying on a single high-stakes practical examination, EFPE uses repeated assessment points, meaningful feedback, and authentic workplace-based problem-solving to support continuous learning and progressive development. This reflects the principles of programmatic assessment, where assessment information and feedback from multiple data points are accumulated over time to support both learning and high-stakes decisions, rather than relying on isolated examinations. Similarly, the Ottawa 2020 Consensus emphasizes that high-stakes decisions should not depend on a single assessment event and that assessment should function across a continuum of stakes rather than a strict separation between formative and summative approaches [2]. EFPE also aligns with longitudinal assessment, in which shorter, repeated assessments are distributed across a defined period, with opportunities for reflection, feedback, and improvement [3].

By embedding these principles, EFPE shifts assessment from an isolated judgment of performance toward a developmental process that supports knowledge retention, transfer, and lifelong professional learning.

To operationalize these principles, EFPE applies a “Learning by Doing” model in which postgraduate students learn through active participation in authentic tasks that address real educational needs within their own institutions. Students are given an extended period—six months in our pilot implementation—to plan, implement, and evaluate a practical project, while engaging with stakeholders, receiving structured feedback, and reflecting on the impact of their work [ 1].

The EFPE model reflects the principles of experiential learning (EL), in which learning occurs through doing, reflection, and adaptation rather than through passive knowledge acquisition. This strengthens the connection between theory and workplace practice, encourages learners to take ownership of their learning, and promotes motivation, accountability, and long-term retention [4–7].

Against this background, EFPE may offer an alternative to traditional postgraduate practical examinations by providing a more authentic, developmental, and institutionally relevant assessment approach. This is particularly relevant in health professions education, where practical training and workplace-based learning are central components of professional development. Accordingly, the current study aims to critically examine the implementation of EFPE across medical schools and to explore its educational value, its impact on learners and institutions, and areas for further improvement.

The study raises the following research questions:

- How do students perceive EFPE?
- What are the direct and indirect effects of the exam on students and institutions?
- What are the recommendations for further use of the exam in different disciplines?

## Methodology

### Context

Medical education is gaining recognition as a distinct specialty within medical schools. The functions of a medical education department include staff research, teaching, and career development. In Egypt, the first academic department of medical education was established at the Faculty of Medicine, Suez Canal University (FOM, SCU) in 2001 [8]. The department offers both on-campus and online academic programs [9]. The on-campus degrees are MSc and MD programs in Medical Education. The candidates enrolled in the on-campus programs are affiliated with different medical schools in Egypt and the Arab region.

### Extended functional practical exam in the Medical Education Department, Suez Canal University (MED-SCU)

**Fig. 1:**
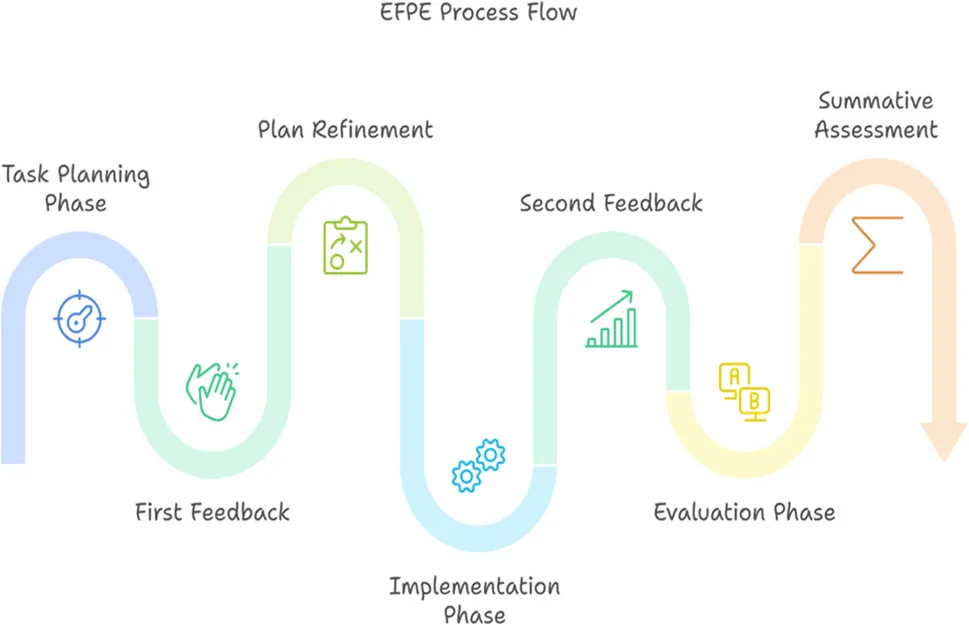
Phases of the Extended Functional Practical Exam (EFPE). This figure illustrates the three phases of the EFPE: planning, implementation, and evaluation. Candidates develop an action plan based on institutional needs, implement it with stakeholder involvement, and evaluate its impact. The process includes two formative feedback sessions and concludes with a final report, an oral presentation, and a summative assessment by both internal and external assessors

The EFPE has three sequential phases, as shown in Fig. 1: two feedback sessions and a summative assessment. Phase 1 is the Planning phase. The candidate must plan the task in writing after receiving a written task that includes all specifics and durations. The task should address institutional needs through a situational analysis that results in three top priorities. Then, the supervisory board selects one of the topics to avoid repetition among students from different institutions. The plan must be submitted within a certain duration (two weeks in the six-month format) after receiving the task. Feedback 1: The examinee receives verbal and numerical feedback from multiple supervisory board members one week after submission. The candidate then refines the plan, taking into account the supervisory board’s review comments.

Phase 2: The implementation phase begins immediately and lasts three months, with full documentation of every step. All stakeholders, including students, peer faculty, administration, and the community (if involved), are involved. Feedback 2: After completing the implementation phase, the examinee receives verbal and numerical feedback one week after submission from the same supervisory board.

Phase 3: The evaluation phase starts to assess the immediate effect. The assessment begins after a defined duration—two months in the six-month format—following the completion of the implementation phase. Finally, a summative assessment is conducted by three internal and external assessors who are different from the supervisors who provided feedback. The candidate submits a final written report that includes all evidence and documentation from the three phases and then delivers a 15-minute oral presentation attended by multiple assessors. The assessors' scores are averaged, and a collective score is given to the candidate on the final report and presentation. To enhance consistency and reduce subjectivity during the Evaluation Phase, predefined evaluation criteria were used rather than a separate formal standardized rubric. The evaluation criteria for the EFPE are summarized in Table 1. Both verbal and numerical feedback were provided across all phases using the same assessment framework. Individual assessor scores were discussed when needed and then averaged to reduce individual bias and improve fairness and reliability. The use of multiple assessment points across the three phases further strengthened the objectivity and consistency of the evaluation process.

**Table 1:** Evaluation criteria for the EFPE

Domain	Description
Relevance to institutional needs	Alignment of the project with identified institutional priorities
Planning quality	Clarity, feasibility, and coherence of the action plan
Implementation process	Effectiveness and organization of execution
Stakeholder involvement	Engagement of relevant stakeholders during implementation
Evidence of immediate impact	Demonstration of measurable or observable outcomes
Sustainability	Potential for continuation and long-term impact
Documentation	Completeness and quality of written records and reporting
Oral presentation performance	Clarity, organization, and effectiveness of the final presentation

### Study design

**Fig. 2:**
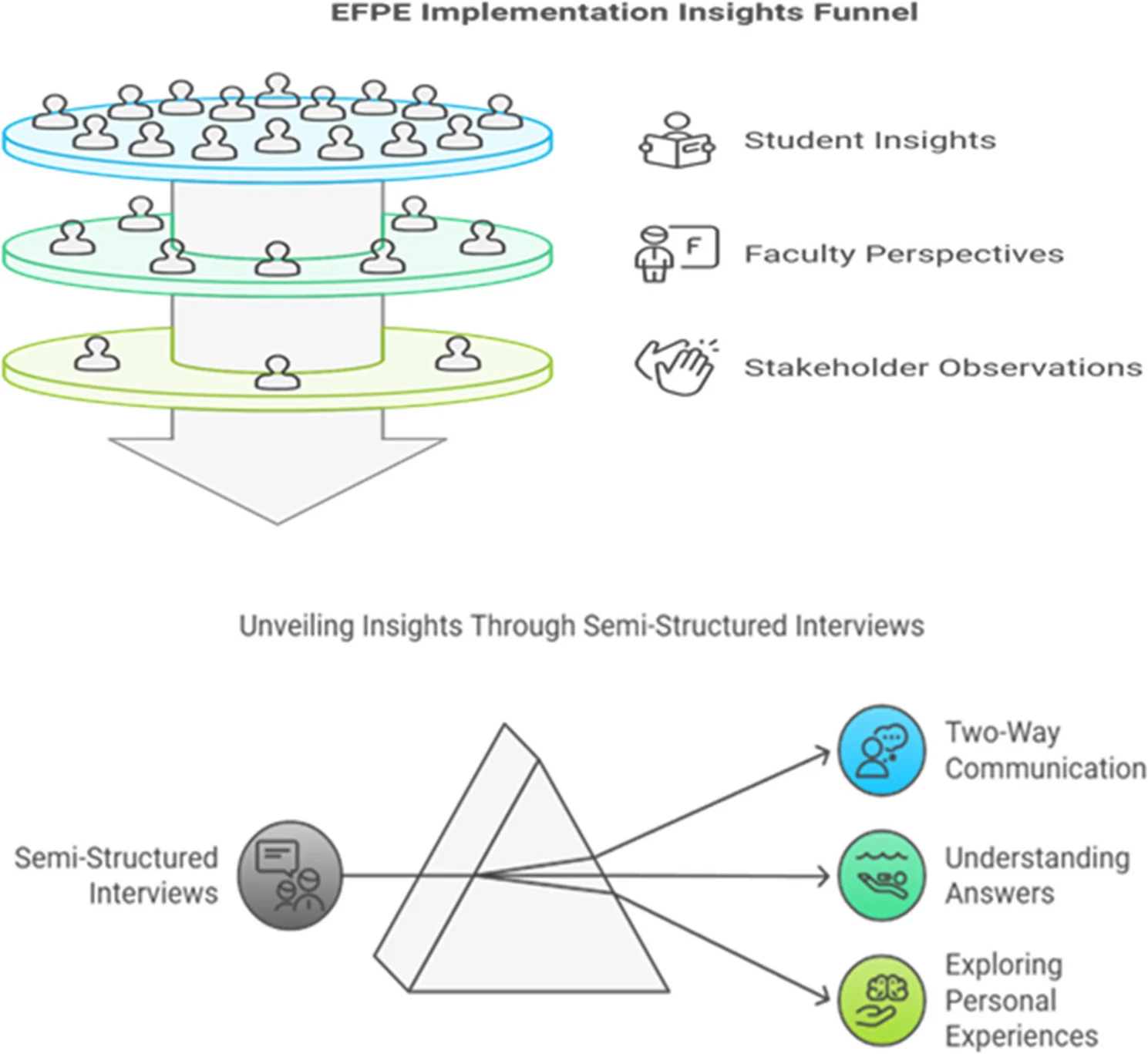
Study design. This figure outlines the design of a reflective qualitative study examining the implementation and impact of EFPE across medical schools. Participants included postgraduate students, faculty involved in supervision and assessment, and institutional stakeholders. Data were collected through semi-structured interviews exploring perceptions of effectiveness, challenges, and areas for improvement

A reflective qualitative study involved different medical schools, as shown in Fig. 2. The data were collected and reported in accordance with the Consolidated Criteria for Reporting Qualitative Research (COREQ) checklist [10].

### Sample

Three samples were included in the study to answer the research questions and provide multiple perspectives on the implementation and impact of the EFPE. A qualitative sampling approach was adopted to ensure depth of understanding rather than numerical representation, with participants selected for their direct experience and involvement in the EFPE process.

Sample 1: A convenience sample of the postgraduate students (PG) who applied for the on-campus MSc and MD programs at FOMSCU-MED from 2019 to 2024. Nine PG students affiliated with five medical schools participated in the study. They have been involved in the EFPE and have completed its phases. The total number of students who completed the EFPE was 14. Accordingly, the response rate among PG students is 64.2%. This sample size was considered appropriate because it included the majority of eligible students who had completed the full EFPE process and provided sufficient depth to capture diverse experiences across different institutions and academic stages.

Sample 2: A purposive, convenience sample of faculty members who were directly involved in implementing and evaluating the EFPE. Three of five faculty members, with a response rate of 60%, were identified and invited to participate in the study. This sample size was considered adequate because these participants were the key faculty directly responsible for feedback, supervision, and assessment decisions, allowing detailed exploration of faculty perspectives on the design, implementation, and quality assurance of the EFPE.

Sample 3: A purposive, convenience sample of medical school stakeholders. The stakeholders were selected based on their observations of the EFPE’s direct and indirect effects on students and institutions. Two stakeholders were chosen, representing two schools with a high number of affiliated PG students who have taken this exam. Given the small total population of faculty directly involved in EFPE. The stakeholder interviews focused on institutional perspectives rather than individual variation.

Participant diversity was ensured by including three participant groups: postgraduate students, faculty examiners, and institutional stakeholders. Participants were recruited from five public medical schools, representing different academic levels and institutional contexts. This diversity allowed the study to capture multiple perspectives on the implementation and impact of the EFPE. The inclusion of students, faculty members, and stakeholders also supported triangulation of perspectives and enhanced the credibility of the findings.

To reduce potential sampling bias associated with purposive and convenience sampling, participants were recruited from multiple institutions and participant groups. In addition, investigator triangulation was applied during thematic analysis through independent coding and regular author discussions. Reflexivity was maintained throughout the study, with interviewers keeping reflective notes to acknowledge potential preconceptions and minimize researcher influence, thereby supporting a balanced and transparent interpretation of the findings.

The adequacy of the sample size was guided by the relevant and direct information participants hold in relation to the study aim. In the present study, participants were selected from three groups with direct experience of the EFPE: postgraduate students who completed the examination, faculty members involved in its supervision and assessment, and institutional stakeholders who observed its impact. The sample’s specificity, the study’s focused aim, and the richness of the interview data supported the adequacy of the sample size.

### Data collection

The insights from the three samples were collected through semi-structured interviews. This qualitative tool encouraged two-way communication. It provided an opportunity for interviewers to learn the answers to questions and the reasons behind the answers and allowed respondents time to open up about personal experiences.

An interview guide (Supplementary Material 1) was developed and conceptualized based on the research objectives. The purpose and questions were tailored according to the three study samples. The guide was reviewed by three medical education experts for clarity, relevance, comprehensiveness, and alignment with the study objectives before data collection.

Ethical approval was obtained from the Research Ethics Committee (REC) of the Faculty of Medicine, Suez Canal University (Research 5689#_2024). The purpose of the study was communicated in the email invitation to all participants. The participant information sheet and informed consent form were also shared by email. Follow-up reminders were sent through WhatsApp to facilitate communication and schedule the interviews. Students who agreed to be included in the study were to sign a written informed consent as acceptance. A detailed information sheet was sent to the participants explaining the purpose of the research, potential benefits and risks, and ensuring that participation was voluntary and that they had the right to refuse participation or withdraw without any reasons or negative consequences. Confidentiality was ensured by keeping the data anonymous and avoiding mentioning any identifying features of the participants. One of the author’s contact details was available for the participants to provide any needed clarification.

The semi-structured interviews were conducted individually, and no non-participants were present during any interview session, which was conducted via synchronous meetings on Microsoft Teams^®^. Each participant took part in a single interview session, and no repeat interviews were conducted. Interviews lasted approximately 30–45 min.

Two research assistants (EA and NW) facilitated these interviews, each facilitating separate sessions. The interview facilitators were female medical education academics, Associate Professors, with postgraduate qualifications; all held MBBCh, MSc, and PhD degrees in Medical Education and had experience in qualitative educational research. They also had prior experience conducting interviews and were familiar with some of the participants through academic and professional contexts related to postgraduate medical education and EFPE implementation, which enabled them to encourage open discussion and made it easier for participants to share their insights. To enhance reflexivity, the facilitators maintained reflective field notes during and after interviews to document their assumptions, potential influence on participants’ responses, and observations about the interview context. During analysis, these reflections were discussed among the research team to minimize individual bias and support a balanced interpretation of both positive and critical views.

### Data analysis

All semi-structured interviews were audio-recorded and transcribed anonymously by the authors. The authors checked the transcripts for accuracy; unclear data were identified and excluded. To maintain participant confidentiality while enabling traceability of quotations, each transcript was assigned a unique identifier comprising the sample number (S), the abbreviation of the participant’s institution (e.g., SCU = Suez Canal University; AU = Alexandria University), and randomly assigned letters (e.g., S2SCUSF) to preserve anonymity. Transcripts were not returned to participants for member checking, comment, or correction, and participants were not asked to provide feedback on the final themes.

**Fig. 3:**
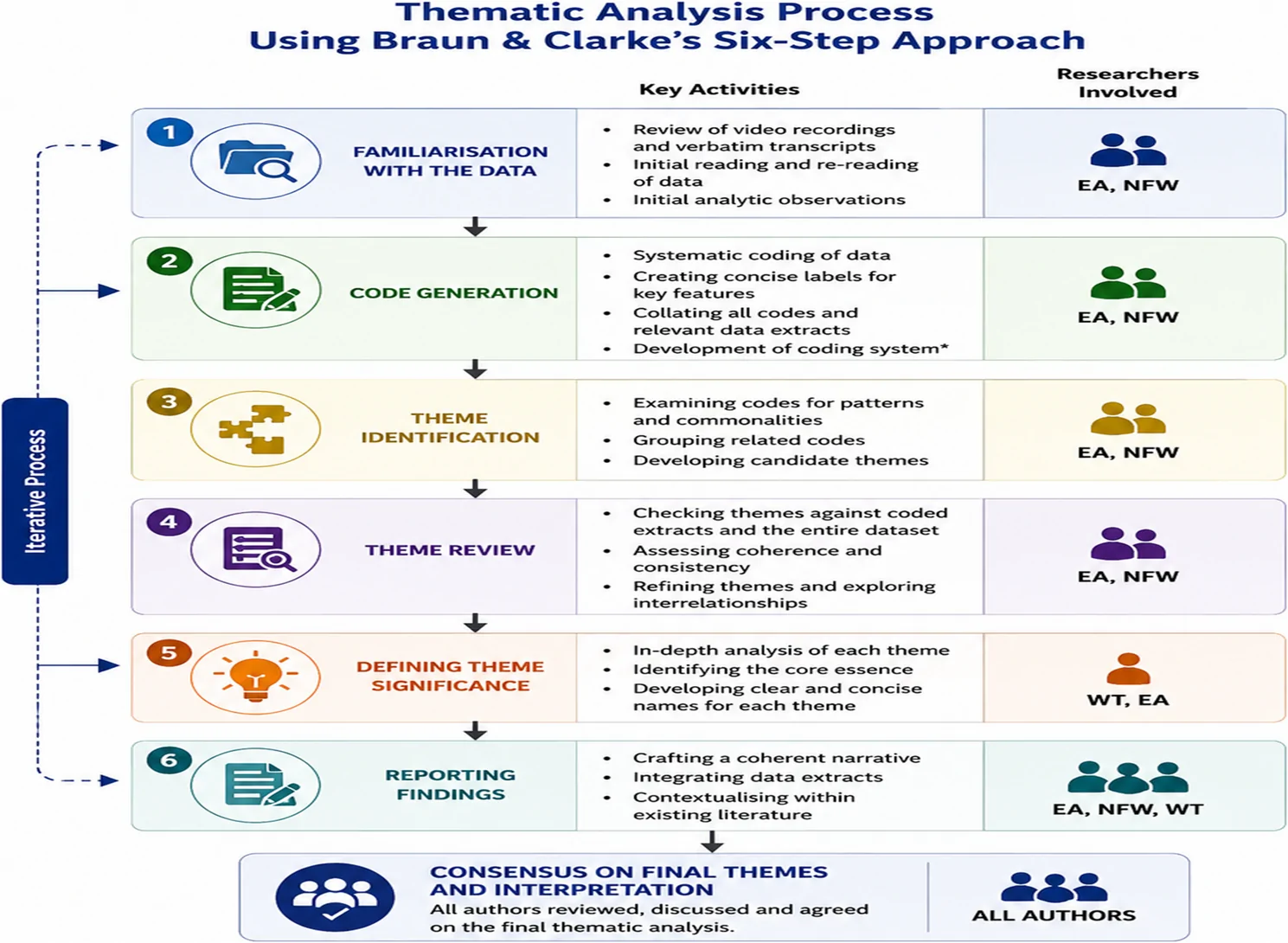
Thematic analysis process using Braun and Clarke’s six-phase approach

The moderator’s field notes and reflections on the transcribed data were attached to provide context. The authors used Braun and Clarke’s six-step process [11] as a coding framework to reach a consensus and ensure a unified interpretation of the data, as shown in Fig. 3. Initial codes were generated manually from meaningful segments of the transcripts and then grouped into related subthemes and overarching themes. Examples of initial codes included learning by doing, formative feedback, institutional relevance, confidence building, workload burden, and the need for a clearer structure.

## Results

### Demographic characteristics

The demographic characteristics of the study participants are as follows: Nine postgraduate students, one (11%) male and eight (89%) females, represented five (100%) public medical schools in the study. Among the Faculty members, the three who participated were female internal examiners. Two stakeholders participated. One (50%) male and one (50%) female. Both represented two out of five (40%) public medical schools.

The coding process moved from initial codes to subthemes and then to overarching themes. Themes were inductively derived from the data. Data analysis from samples S1, S2, and S3 under the classified themes revealed several themes and subthemes, as shown in Table 2. These include three main themes: format and differences from traditional exams, the impact of the EFPE and its challenges, and areas for improvement.

**Table 2:** Emergent themes and subthemes identified through thematic analysis of semi-structured interviews

Themes	Subthemes
EFPE Format and Difference from Traditional Examinations	• Practical, Structured, and Longitudinal Assessment • Feedback, Mentorship, and Objectivity • Institutional Relevance, Sustainability, and Administrative Support • Student Involvement, Duration, and Level of Implementation
Impact and Strengths of EFPE on Students	• Professional and Transferable Skills Development • Confidence Building and Professional Identity • Preparation for Future Academic and Professional Roles • Variability Based on Student Engagement
Implementation Challenges and Enablers	• Stress and Workload • Resource Demands • Topic Selection Challenges • Need for Clearer Structure

1. EFPE Format and Difference from Traditional Examinations

Participants first described how EFPE differs from traditional postgraduate practical examinations, particularly in its practical structure, longitudinal design, feedback process, and stronger alignment with workplace and institutional needs. Most postgraduate students, all faculty members, and both stakeholders emphasized that EFPE offered a more authentic, structured, and workplace-based assessment experience compared with traditional single-point examinations.

1.1  Practical, Structured, and Longitudinal Assessment

Across all participant groups, EFPE was described as a practical and meaningful assessment that reflects real institutional needs and workplace responsibilities. Participants valued its hands-on nature and its focus on solving actual institutional problems rather than relying on theoretical recall alone. The structured and extended nature of the exam allowed students to plan ahead, organize their work, and gradually mature through repeated engagement and reflection. Unlike traditional single-point examinations, EFPE was viewed as a process-focused assessment where students learn while being assessed.

- S1SCUHL: *“More practical and related to work*,* structured and beneficial to the students.”*
- S1SUHO: *“Lengthening the exam over a long duration helps me learn by doing.”*
- S2SCUDK: *“Hands-on experience that promotes the application of knowledge.”*
- S2SCUNH: *“It improves the objectivity of the exam*,* includes feedback*,* longitudinal assessment*,* increases confidence*,* and enhances the application of knowledge.”*
- S3SUSA: *“Focus on practical skills rather than theoretical development*,* objective exam*,* solve institutional need*,* provide students with many skills.”*
- S3SCUOL: *“It is a process evaluation rather than an outcome evaluation which will ensure the development of the student and assessment for learning.”*

1.2  Feedback, Mentorship, and Objectivity

Feedback and mentorship were viewed as central strengths of EFPE. Participants appreciated receiving constructive feedback across multiple stages, which supported motivation, confidence, and continuous improvement. The opportunity to revise work based on feedback helped students feel that the assessment was developmental rather than judgmental. In addition, the use of multiple assessors and practical performance tasks strengthened perceptions of fairness and objectivity compared with traditional oral and practical examinations.

1.3  Institutional Relevance, Sustainability, and Administrative Support

Participants strongly valued the alignment of EFPE tasks with institutional priorities. Working on projects that addressed real workplace challenges created immediate benefits for their organizations and increased motivation. Sustainability was considered essential, particularly when projects were embedded into departmental policies or institutional bylaws. Administrative involvement was also viewed as important for facilitating implementation, providing resources, and ensuring continuity beyond the examination itself.

- S1SUHO: *“My project was an institutional need that guaranteed its sustainability even after the end of my exam.”*
- S2SCUSF: *“The sustainability of the task is important to have an authentic effect on the institution.”*
- S3SCUOL: *“I included it in the new postgraduate bylaws. It is used now in different departments.”*
- S3SUSA: *“Involvement of the higher administration in the selection of the task… they facilitate the implementation.”*

1.4  Student Involvement, Duration, and Level of Implementation

Participants also reflected on practical considerations for future EFPE implementation. Greater student involvement in topic selection was seen as important for improving motivation and ownership while maintaining alignment with institutional needs. Views on duration varied; some considered six months sufficient, whereas others recommended a longer or more flexible timeframe depending on task complexity. Some faculty participants also suggested that EFPE may be more suitable at the PhD level to ensure learner readiness and avoid overlapping with thesis requirements. 

While participants valued the structure and process of EFPE, they also emphasized its broader impact on their personal and professional development. The second theme, therefore, explored how EFPE influenced students’ skills, confidence, and readiness for future academic and leadership roles.

2. Impact and Strengths of EFPE on Students

Beyond the exam format itself, participants highlighted EFPE’s significant educational and professional impact on students. Most postgraduate students, all faculty members, and both stakeholders reported noticeable improvements in communication, leadership, project management, confidence, and preparation for future academic roles, such as PhD studies and institutional leadership responsibilities.

2.1  Professional and Transferable Skills Development

Participants consistently reported that EFPE enhanced a wide range of professional and transferable skills, including communication, academic writing, presentation skills, project management, leadership, critical thinking, and planning. Through report writing, presentations, stakeholder engagement, and practical implementation of projects, students developed stronger verbal and written communication as well as better organizational and managerial abilities. Many participants also highlighted leadership growth, including negotiation, resource allocation, problem-solving, and conflict management. These experiences contributed to a broader improvement in knowledge, skills, and attitudes (KSAs), supporting deeper understanding and better application of learning in workplace settings.

- S1SCUHA: *“Written reports helped to improve academic writing and oral communication skills through presentation.”*
- S1SCUHA: *“Leadership skills*,* resource allocation*,* and negotiation skills.”*
- S2SCUSF: *“It improves their academic writing skills. Planning is the main gain from this exam.”*
- S3SCUOL: *“Help to develop a generation capable of solving problems*,* long-term effect of developing a strategic way for solving problems.”*

2.2  Confidence Building and Professional Identity

EFPE significantly increased students’ confidence in applying practical skills and presenting their work. Participants described feeling more capable, independent, and prepared to face academic and professional challenges. Repeated practice, feedback, and successful implementation of projects helped students overcome fear and uncertainty while improving confidence in oral presentations, teamwork, and professional decision-making. This strengthened their sense of professional identity and self-efficacy.

- S1SCUHL: “*It changed me after I completed it. I trust myself and my capabilities.”*
- S2SCUNH: *“Increase confidence by trying and failing. Increase confidence by a deeper understanding of the theoretical background.”*
- S3SUSA: *“Boosts their confidence and research skills.”*

2.3   Preparation for Future Academic and Professional Roles

Participants felt that EFPE effectively prepared them for future academic progression, particularly PhD studies, and for broader leadership roles within their institutions. The process helped students develop a stronger academic foundation, improve planning and research skills, and approach future professional tasks with greater readiness. Several participants described EFPE as a strong foundation for lifelong professional development and institutional leadership.

- S1SCUHL: *“It prepared me for a PhD*,* and I can do any future tasks.”*
- S2SCUSF: *“It improves academic writing skills. Planning is the main gain from it “*.
- S3SCUOL: *“Help to develop a generation capable of solving problems.”*

2.4  Variability Based on Student Engagement

Participants noted that the impact of EFPE was influenced by individual student effort, motivation, and responsiveness to feedback. Students who were enthusiastic, internally motivated, and actively engaged in the process appeared to gain greater benefit from the experience, whereas superficial engagement limited the exam’s developmental value.

- S1SUHO: *“I was enthusiastic and internally motivated in each step that helped me overcome obstacles.”*
- S2SCUSF: *“It depends on the learner being enthusiastic or responding to feedback.”*
- S3SUSA: *“But the level of change depends on the effort and personality of the student.”*

Despite these strengths, participants also identified several challenges related to stress, workload, and implementation barriers. These concerns shaped the third theme, which focused on practical challenges and recommendations for improving the sustainability and effectiveness of EFPE.

3. Implementation Challenges and Enablers

Despite the positive impact of EFPE, participants also identified several practical and emotional challenges related to implementation. Most postgraduate students, all faculty members, and both stakeholders discussed issues related to stress, workload, resource demands, clarity of expectations, and topic selection.

- Stress and Workload

- S1SCUHA: *“I felt it was a burden at the start and stressful due to other commitments and being close to the exam.”*
- S2SCUNH: *“Stressful at the start and while waiting for the feedback*,* multiple phases and decisions.”*

- Resource Demands

- S2SCUDK: *“Burden on students*,* resource demanding in terms of staff time and effort.”*

- S1AUHAH: *“It was new*,* challenging*,* but it was not clear how I would accomplish. I was scared of assessing impact through this short period.”*

- Topic Selection Challenges

- S2SCUDK: “Topics may be repeated with time that affect the quality of the experience.”

### Development of a preliminary implementation framework

Based on an interpretive synthesis of the study findings and the existing design features of EFPE, we developed a preliminary four-level framework to organize the factors that participants identified as relevant to EFPE implementation. The framework was developed through an interpretive synthesis of the themes and subthemes identified in qualitative analysis. Rather than representing four additional themes, the framework reorganizes the empirical findings according to the level within the EFPE implementation system at which each factor primarily operates. The framework is proposed as an empirically informed working model that requires further validation across different educational and institutional contexts.

Level 1: Project Design was primarily informed by the theme “EFPE Format and Difference from Traditional Examinations,” particularly the subthemes Practical, Structured, and Longitudinal Assessment and Student Involvement, Duration, and Level of Implementation, together with findings from the Challenges and Enablers theme relating to Topic Selection Challenges and Lack of Clarity and Expectations. These findings highlighted the importance of authentic project selection, alignment with institutional priorities, clear implementation plans, appropriate timelines, and structured expectations as core design features of EFPE.

Level 2: Learner Role was derived mainly from the theme “Impact and Strengths of EFPE on Students,” particularly the subthemes Professional and Transferable Skills Development, Confidence Building and Professional Identity, Preparation for Future Academic and Professional Roles, and Variability Based on Student Engagement. Additional support came from the subtheme of Student Involvement, Duration, and Level of Implementation, which emphasised learner ownership through participation in topic selection. Together, these findings identified learner engagement, motivation, responsiveness to feedback, self-directed learning, and professional growth as central components of successful EFPE implementation.

Level 3: Departmental Support System was informed predominantly by the subtheme Feedback, Mentorship, and Objectivity within the EFPE Format and Difference from Traditional Examinations theme, together with the Challenges and Enablers subthemes Stress and Workload, Resource Demands, and Lack of Clarity and Expectations. These findings demonstrated that effective implementation requires structured supervision, formative feedback, transparent assessment processes, faculty guidance, communication mechanisms, and adequate departmental resources.

Level 4: Institutional Environment was primarily derived from the subtheme Institutional Relevance, Sustainability, and Administrative Support within the EFPE Format and Difference from Traditional Examinations theme. This was reinforced by participants’ recommendations regarding stakeholder engagement, sustainability, and institutional commitment. These findings indicated that successful implementation depends on an enabling organisational environment characterised by administrative support, alignment with institutional priorities, stakeholder engagement, resource allocation, and policies that facilitate sustainability.

Finally, the cross-cutting principles emerged from concepts that recurred across multiple themes, subthemes, and participant groups rather than belonging to a single implementation level. Authenticity and longitudinal learning reflected the overall structure of EFPE described throughout the EFPE Format and Difference from Traditional Examinations theme. Feedback and mentorship linked the learner and departmental levels, while stakeholder engagement and sustainability connected departmental and institutional processes. Quality assurance and assessment for learning reflected overarching principles underpinning the implementation and educational value of EFPE across all four levels.

**Fig. 4:**
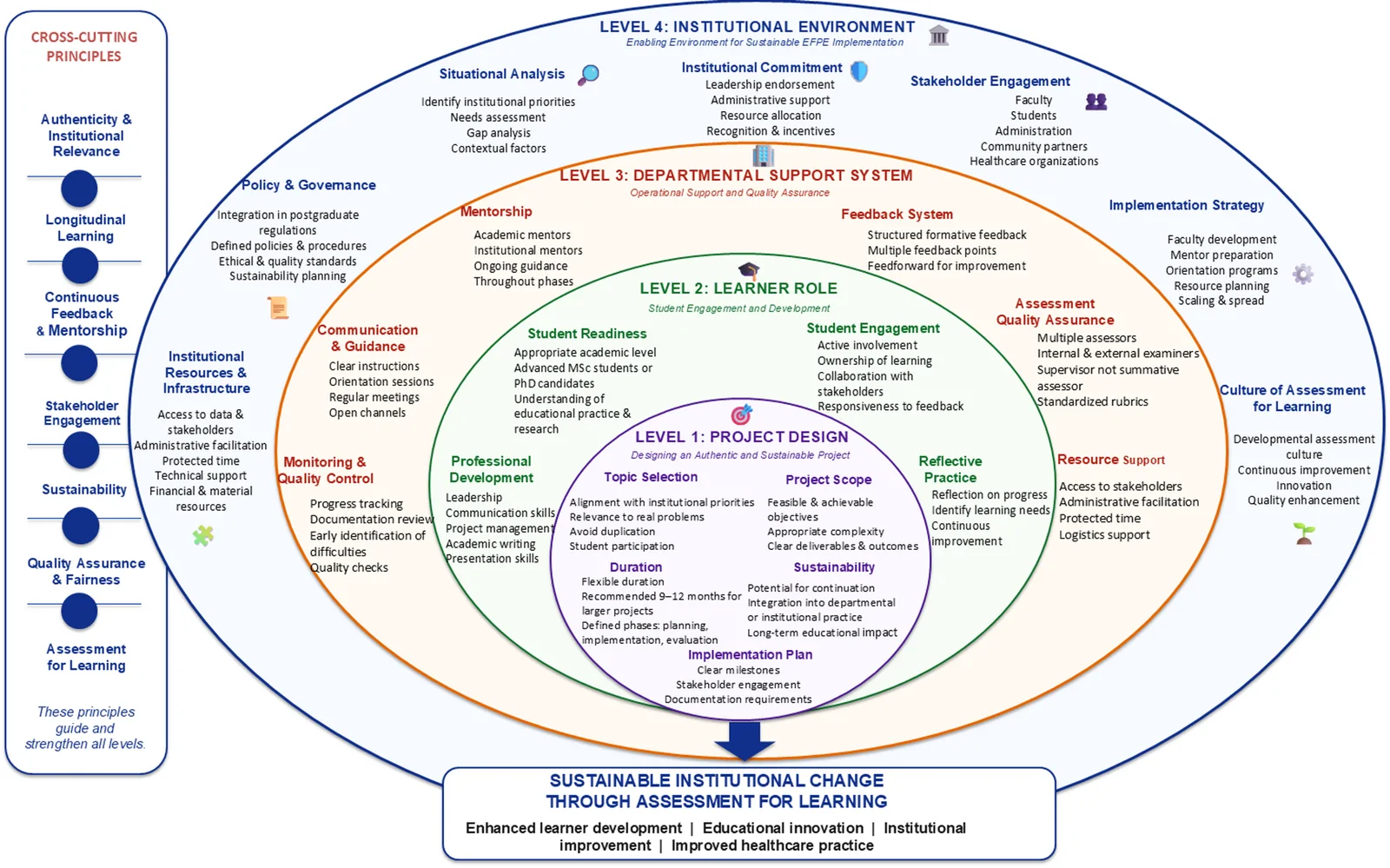
A four-level integrated framework for implementation of the extended functional practical examination (EFPE)

Figure 4 therefore presents a preliminary, empirically informed implementation framework. The four interconnected levels organize factors identified by participants as relevant to implementation, while the cross-cutting principles reflect concepts recurring across themes and participant groups.

## Discussion

The current study critically examined the implementation of EFPE across different medical schools and explored its effectiveness as a postgraduate assessment model. Participants identified both benefits and challenges, but overall reflections showed strong engagement and positive perceptions of the process. EFPE was frequently described as more practical, structured, and closely aligned with workplace realities than traditional postgraduate practical examinations. Its longitudinal design, repeated feedback, and authentic institutional tasks support both learning and performance development.

Building on this, participants particularly valued the practical application embedded within EFPE, which helped bridge the gap between theoretical knowledge and real-world practice. Participants described it as a more hands-on and work-related approach that aligns learning with institutional needs and immediate workplace relevance. Through active involvement and experiential learning, students engage in authentic tasks that foster deeper understanding, knowledge retention, and professional development rather than merely preparing for examinations. This aligns with Kolb’s experiential learning theory, which describes learning as a process where knowledge is created through the transformation of experience and reflection [12].

In addition to its practical value, participants also highlighted the importance of fairness and objectivity within the assessment process. Participants noted that EFPE provides a more objective assessment compared to traditional examinations. The involvement of multiple assessors and practical tasks reduces subjectivity and supports fairer evaluation. During the final Evaluation Phase, all assessors used the same structured assessment criteria across predefined domains, and the final assessment was conducted by three internal and external assessors who were different from the supervisors involved in formative feedback. Scores were averaged to reduce individual bias and improve reliability. Although objectified methods do not inherently guarantee greater reliability [13], EFPE strengthens reliability through multiple assessment points, repeated feedback opportunities, and the involvement of several assessors rather than relying on a single isolated examination event.

A major factor contributing to student development was the role of structured feedback and mentorship throughout the EFPE process. Interactions with supervisors provided personalized guidance that enhanced critical thinking, problem-solving, communication, and confidence. EFPE successfully closes the feedback loop by ensuring that feedback translates into actionable improvement rather than remaining a passive process. Meaningful feedback is well established as a driver of student learning [14], although implementing effective feedback processes remains challenging in higher education [15]. In EFPE, feedback serves as a developmental process in which learners use comments to improve future performance and learning strategies, thereby strengthening both short-term achievement and professional growth [16, 17].

This developmental process was reflected in participants’ descriptions of their progression as learners over time. Initial stress and uncertainty gradually shifted into greater awareness of learning needs, improved confidence, and a stronger professional identity. This aligns with the Stages of Competence Model [18], in which learners progress from conscious incompetence to early conscious competence through repeated feedback, reflection, and authentic workplace engagement. While the current study primarily captured immediate professional growth and short-term application during the six-month assessment period, EFPE provides a strong foundation for sustained competence development and, for some learners, may support further progress toward unconscious competence, depending on engagement and performance [19].

Unlike shorter active learning strategies such as case discussions or simulations, EFPE extends learning through authentic workplace implementation over time, requiring learners to apply, evaluate, and sustain change within real institutional settings. This longitudinal process supports early progression toward conscious competence and provides a stronger foundation for sustained professional development beyond single-event assessment experiences.

Repeated documentation, report writing, presentations, and stakeholder engagement also strengthened verbal and written communication skills, helping students articulate complex ideas clearly and confidently in professional settings. These experiences supported confidence-building and helped students recognize their own achievements and professional growth. Through this process, EFPE integrates reflection in and on action [20] and experiential learning [12], encouraging learners to take responsibility for their learning and apply knowledge within authentic workplace contexts. Together, these findings are reflected in Level 2 (Learner Role) of the preliminary framework, which highlights learner agency and active participation as important influences on the EFPE process, while recognising that the relationship between learner engagement and educational outcomes requires further investigation.

Despite these benefits, participants described the emotional demands of the process, particularly during the early stages of EFPE. Many participants initially experienced high levels of stress due to workload, uncertainty, and proximity to examinations. This anxiety can act as both a challenge and a motivator. High levels of test anxiety may negatively affect academic performance [21] while moderate anxiety may encourage stronger preparation and engagement [22]. These findings suggest that while stress may initially challenge students, it can also stimulate professional growth when appropriately supported.

To reduce cognitive overload and prevent student burnout, EFPE was intentionally designed as a phased longitudinal assessment rather than a single high-stakes examination. The three-stage structure allowed students to distribute the workload over time, receive formative feedback before the final evaluation, and progressively refine their work rather than face a single summative judgment. Continuous mentorship and supervisory support also helped students manage uncertainty and workload, while selecting tasks aligned with institutional needs reduced duplication between assessment requirements and workplace responsibilities. Participants further suggested greater flexibility in duration based on task complexity, which may enhance feasibility and reduce stress in future implementation.

To reduce this stress and improve sustainability, the institutional relevance of tasks emerged as an important strategy. One important EFPE strategy for managing student anxiety involves selecting tasks based on institutional needs. This creates urgency and relevance while helping students secure administrative support for implementation and sustainability. Students, therefore, become active agents of institutional change rather than isolated learners completing an academic requirement. Stakeholder participation is widely recognized as a critical factor for successful organizational change [23], and EFPE strengthens this process by aligning student work with institutional priorities.

Similarly, participants emphasized that greater involvement in topic selection could further strengthen motivation and ownership of learning. Participants suggested involving both students and administration in topic selection to ensure tasks are relevant and motivating. Student involvement in selecting learning tasks enhances intrinsic motivation and ownership of learning [24], while learner-centered assessment supports empowerment and self-regulated learning [25]. Clear expectations and relevance are equally important for reducing anxiety and improving engagement, as understanding assessment expectations improves motivation, performance, and participation [26]. These findings are reflected in Level 1 (Project Design) of the preliminary framework, which positions careful project design as an important foundation for EFPE implementation, although further research is needed to examine how these design features influence implementation and outcomes across different contexts.

Although EFPE requires significant faculty time, particularly for the two formal feedback sessions and continuous supervision, participants considered this investment educationally valuable because it supports meaningful feedback, stronger learner development, and more sustainable institutional change compared with single-point examinations. The benefit extends beyond student assessment, as many projects directly address institutional needs and contribute to long-term educational improvement.

The preliminary framework provides a multilevel perspective for understanding how these workload and resource demands might be addressed. In particular, the interaction between Level 3 (Departmental Support System) and Level 4 (Institutional Environment) suggests that the sustainability of EFPE should not depend solely on the efforts of individual faculty members. At the departmental level, structured supervision, shared feedback and assessment responsibilities, clear communication, and appropriate workload distribution can support the operational delivery of EFPE. However, these mechanisms require an enabling institutional environment that provides leadership support, resource allocation, alignment with institutional priorities, supportive policies, and broader stakeholder engagement. This is consistent with organisational change literature emphasising that successful and sustainable implementation depends on the interaction between organisational capacity, leadership support, resource availability, and stakeholder engagement [23]. The interaction between these two levels may therefore distribute responsibility across the educational system, reduce dependence on individual faculty members, and support more feasible and sustainable implementation.

However, this interaction may be particularly challenging in institutions with larger student cohorts or less flexible administrative structures. Limited faculty availability, restricted supervisory capacity, and difficulty aligning projects with institutional priorities may reduce feasibility. Strategies such as assigning institutional mentors, involving alumni as ambassadors, using group supervision models, and prioritizing projects with clear institutional relevance may help improve scalability and reduce faculty burden while preserving educational quality.

### Limitations & future research

Despite the methodological rigor of the study, several limitations should be acknowledged. The relatively small, self-selected sample may not fully reflect the broader range of perspectives among postgraduate students, faculty members, and stakeholders involved in similar programs. Participants who had stronger or more positive experiences with EFPE may have been more likely to participate, introducing potential selection and response bias. While purposive and convenience sampling are appropriate for qualitative inquiry, they limit the transferability and generalizability of the findings to broader postgraduate education contexts. This limitation is particularly relevant given that EFPE is a relatively new assessment tool and has only been recently implemented.

Although several strategies were employed to enhance trustworthiness, including investigator triangulation, iterative theme review, and consensus among authors regarding final themes and interpretations, transcripts were not returned for member checking and themes were not validated with participants, which may have limited confirmability.

Additionally, although semi-structured interviews are well-suited to capturing rich, detailed experiences, they are inherently susceptible to interviewer influence and participant interpretation. Two members of the research team who conducted interviews had prior familiarity with the implementation of the EFPE and with some participants. This may have introduced advocacy and social desirability biases, potentially increasing the likelihood of favorable responses. To mitigate this risk, participants were assured of confidentiality and encouraged to share both positive and negative experiences; however, such influences cannot be entirely excluded.

Furthermore, this study explored participants’ perceptions and experiences of the EFPE and relied primarily on qualitative data. Consequently, the findings should be interpreted as participants’ reported views of the program’s contribution to their learning and professional development rather than as evidence of actual educational effectiveness or objectively measured skill development. The absence of objective performance data and complementary quantitative outcome measures limits the ability to evaluate measurable educational outcomes, compare the EFPE with traditional assessment approaches, or determine the extent to which perceived benefits translated into sustained improvements in practice.

In particular, competencies such as leadership, conflict resolution, and other professional skills were identified through participants’ reflections and reported workplace experiences rather than through validated assessment instruments or objective performance measures. While these accounts provide valuable insights into perceived professional growth and the potential influence of the EFPE, future studies incorporating independent evaluation methods and objective outcome measures would strengthen the evidence regarding its effectiveness.

Future research is recommended to examine knowledge transfer, learner performance, leadership development, and the broader institutional impact of EFPE, using larger, more diverse samples and mixed methods approaches that combine qualitative insights with quantitative evaluation. As the current study did not include a comparison group of students undertaking traditional single-point postgraduate practical examinations, the ability to statistically compare EFPE outcomes with conventional assessment models remains limited. Future comparative studies are needed to evaluate differences in skill development, academic performance, and long-term professional outcomes between EFPE and traditional assessment approaches. Research should also explore the application of EFPE beyond postgraduate medical education, particularly across other health professions such as nursing, pharmacy, dentistry, and allied health disciplines, where authentic and longitudinal assessment is equally valuable. Larger-scale studies are needed to assess its feasibility, scalability, faculty requirements, and long-term impact on learner development and professional practice across different institutional and disciplinary contexts.

## Conclusion

EFPE offers a practical, student-centered approach to postgraduate assessment by shifting evaluation from a single high-stakes examination toward a longitudinal, feedback-oriented, and workplace-based learning process. Its emphasis on authentic tasks, structured mentorship, and continuous feedback supports student learning, short-term professional growth, confidence building, and early competence development, while creating stronger alignment between assessment and institutional needs.

The findings highlight several actionable implications for practice and policy. Institutions implementing EFPE should ensure clear supervisory structures, meaningful feedback processes, and assessment tasks that address real institutional priorities while allowing appropriate student involvement in topic selection. Flexibility in assessment duration and stronger mentorship systems may further improve feasibility and sustainability.

At the policy level, EFPE supports the broader move toward programmatic and learner-centered assessment models in health professions education. Educational leaders and policymakers should consider assessment systems that promote continuous development, stakeholder engagement, and workplace relevance rather than relying solely on single-point practical examinations. Such approaches may enhance both learner outcomes and sustainable institutional improvement.

## Supplementary Information

Supplementary Material 1.

## Data Availability

Data is provided within the manuscript or supplementary information files.

## Abbreviations

EFPE: Extended Functional Practical Examination
EL: Experiential learning
FOM-SCU: Faculty of Medicine, Suez Canal University
KSAs: Knowledge, skills, and attitudes
LA: Longitudinal assessment
MED-SCU: Medical Education Department, Suez Canal University
PG: Postgraduate students
REC: Research Ethics Committee
