# Supplementary material for: The extended functional practical examination (EFPE): an innovative model for advancing assessment for learning in postgraduate medical education
Source: BMC Med Educ. 2026 Oct 2;26:1571. doi: 10.1186/s12909-026-10345-0 (PMC13633635; doi:10.1186/s12909-026-10345-0)
Supplement: Supplementary file 1 [file 12909_2026_10345_MOESM1_ESM.docx]

**Semi-Structured Interview Guide**

**Interview Guide for Postgraduate Students.**

**Themes:**

EFPE Format

Impact/ effect of EFPE on the postgraduate students (during and after)

Students’ feedback on EFPE

Suggestions for EFPE improvement

- **EFPE Format**

1. How is the EFPE Format different from the traditional practical exams?
2. What were your initial thoughts and feelings about the EFPE format?
3. How did you get prepared for EFPE?
4. How does the evaluation of EFPE differ from other practical exams?
5. How did your EFPE address institutional needs, improve, or make a change in your institution?

- **Impact/ effect of EFPE on the postgraduate students (during and after)**

6. Being an extended, practical, and functional exam, what do you learn from this format?
7. How did the EFPE impact your knowledge, skills, and attitude during the EFPE phases?
8. How did the EFPE impact your knowledge, skills, and attitude after the EFPE?
9. In what ways did the EFPE affect your understanding and retention of your knowledge?
10. How did the EFPE impact your confidence in applying your knowledge and practical skills?
11. How does EFPE influence your motivation regarding learning?
12. How did the EFPE affect your social life and relationships during the preparation period and the EFPE phases?
13. In what ways do you think the EFPE format could better prepare you for future academic life?
14. How did the EFPE influence your stress levels compared to other practical exams?

- **Students’ feedback on EFPE**

15. From your point of view, what are the strengths and weaknesses of EFPE?

- **Suggestions for EFPE improvement**

16. What changes would you suggest improving the experience of EFPE for students?

**Interview Guide for Faculty Members Involved in EFPE**

**Themes:**

EFPE Format

Impact/ effect of EFPE on the postgraduate students (during and after

Impact/ effect of EFPE on the institution

Suggestions for EFPE improvement

Faculty members ’ feedback on EFPE

- **EFPE Format**

1. How do you believe the **EFPE** format has changed students' preparation for the practical exam?
2. What are your initial thoughts and feelings about the **EFPE** format and its potential impact on students?
3. What is the difference between EFPE and other traditional practical exams?

- **Impact/ effect of EFPE on the postgraduate students**

4. In your opinion, how has the EFPE influenced students' stress levels compared to traditional exams?
5. How do you think the EFPE has affected students' understanding and retention of knowledge?
6. From your perspective, how has the EFPE impacted students' confidence in applying practical skills and knowledge?
7. In your experience, has the EFPE influenced students' motivation to get engaged in learning?
8. How has the EFPE affected students' social lives and relationships during the preparation period and the exam phases, in your view?
9. In what ways do you think the **EFPE** format could better prepare students for future academic life, based on your observations?

- **Impact/ effect of EFPE on the institution**

10. How did EFPE address institutional needs, improve, or make a change in your institution?

- **Feedback on EFPE**

11. From your point of view what are the strengths and weaknesses of EFPE?

- **Suggestions for EFPE improvement**

10. What changes or improvements would you suggest enhancing the experience of the **EFPE** for students, from your perspective as a faculty member?

**Interview Guide for stakeholders.**

Addressing the needs

Impact/ effect of EFPE on the institution

Maintaining the product of the EFPE

Administrative support and EFPE implementation Facilitation

- **Addressing the needs**

How did the EFPE address institutional needs?

- **Impact/ effect of EFPE on the institution**

How does the EFPE improve, or make a change in your institution?

- **Maintaining the product of the EFPE**

How is the effect and the product of the EFPE maintained after each EFPE?

- **Administrative support and EFPE implementation Facilitation**

How did the institution administration support the EFPE and facilitate its implementation?
